# Malaria care seeking behavior of individuals in Ghana under the NHIS: Are we back to the use of informal care?

**DOI:** 10.1186/s12889-015-1696-3

**Published:** 2015-04-12

**Authors:** Ama Pokuaa Fenny, Felix A Asante, Ulrika Enemark, Kristian S Hansen

**Affiliations:** Economics Division, Institute of Statistical, Social and Economic Research (ISSER), University of Ghana, P.O. Box LG 74, Legon LG74 Accra, Ghana; Department of Public Health, Aarhus University, Vennelyst Boulevard 6, 8000, Århus C, Denmark; Department of Global Health and Development, Faculty of Public Health and Policy, London School of Hygiene and Tropical Medicine, 15-17 Tavistock Place, WC1H 9SH London, UK

**Keywords:** Malaria, Health insurance, Care seeking, Household survey, Multinomial logit

## Abstract

**Background:**

Malaria is Ghana's most endemic disease; occurring across most parts of the country with a significant impact on individuals and the health system as whole. Treatment seeking for malaria care takes various forms. The National Health Insurance Scheme (NHIS) was introduced in 2004 to promote access to health services to mitigate the negative impact of the user fee regime. Ten years on, national coverage is less than 40% of the total population and patients continue to make direct payments for health services. This paper analyses the care-seeking behaviour of households for treatment of malaria in Ghana under the NHI policy.

**Method:**

Using a cross-sectional survey of household data collected from three districts in Ghana covering the 3 ecological zones namely the coastal, forest and savannah, a multinomial logit model is estimated. The sample consists of 365 adults and children reporting being ill with malaria in the last four weeks prior to the study.

**Results:**

Out of the total, 58% were insured and 71% of them sought care from a formal health facility. Among the insured, 15% chose informal care compared to 48% among the uninsured. The results from the multinomial logit estimations show that health insurance and travel time to health facility are significant determinants of health care demand. The results show that the insured are 6 times more likely to choose regional/district hospitals: 5 times more likely to choose health centres/clinics and 7 times more likely to choose private hospitals/clinics over informal care when compared with the uninsured. Individual characteristics such as age, education and wealth status were significant determinants of health care provider choice for specific categories of health facilities.

**Conclusion:**

Overall, for malaria care the uninsured are more likely to choose informal care compared to the insured for the treatment of malaria.

## Background

Malaria remains a leading cause of morbidity and mortality in the developing world especially in sub-Saharan Africa with 90 percent of all malaria deaths occurring in the region. In 2012, malaria killed an estimated 482 000 children under five years of age [1]. It is the most significant public health problem in Ghana where it accounts for 38 percent of all outpatient illnesses, 35 percent of all admissions, and 34 percent of all deaths in children under five years [2]. As part of the strategies to achieve the goal of universal access to appropriate interventions for all populations at risk of malaria, it is required that the appropriate clinical assessment is undertaken before treatment with antimalarial [3]. However, one of main barriers to health care access is the direct out-of-pocket payment form of health care financing which pertains in most Sub-Saharan African countries [4]. The ongoing debate is for health sector financial reforms to adopt pre-payment and risk-sharing options [5]. Ghana has done so by introducing a National Health Insurance Scheme in 2005. The main objective of the scheme is to promote access to quality health care services [6]. Since the start of the National Health Insurance Scheme (NHIS) in 2005, overall OPD cases have shown a marked increase, suggesting that the NHI policy has led to an increase in health service usage [7]. In spite of this, coverage of the scheme is still low (about 40 percent of total population) and regressive in nature [8].

The choice of malaria care is affected by various factors including socio-demographic characteristics, family and individual resources and health condition [9-14]. Health insurance can improve access but there is no guarantee that even those with valid membership will use the services provided under the scheme. Relying on the behavioural model [15,16], this paper assesses the determinants of choice of care for malaria treatment in Ghana. The appropriate treatment of malaria and the correct use of antimalarials is needed in order to achieve Ghana’s goal of reducing morbidity and mortality caused by malaria by 75 percent by 2015 [3]. This study was conducted as part of a larger project that investigated the impact of health care financing on health-seeking behaviour, quality of care, efficiency, resource mobilization and health status among households in Ghana and Tanzania.

The next section presents an overview of the health sector and attributes of health providers as well as an overview of the health insurance system in Ghana. This is followed by a description of the methods and results. The final section includes the conclusion and policy implication.

### Healthcare and health insurance in Ghana

Health care delivery in Ghana is provided by both the public and private sectors, with the public sector organized according to hierarchy with teaching hospitals at the national level at the apex, followed by regional hospitals, district hospitals, sub-district (health centres) and community levels - Community-based Health Planning and Services (CHPS). CHPS is an inclusive programme for transforming clinic based primary health care to community-based health services. Health centres and CHPS provide primary care, with district and regional hospitals providing secondary health care as well as primary health care. Tertiary services including specialised clinical care are provided at the teaching hospitals. District hospitals are staffed with one or more qualified medical doctors, nurses, pharmacists, laboratory technicians, auxiliary nurses and other support personnel. Health centers are manned by a medical assistant or a nurse.

Health care financing in Ghana has gone through many dynamics, from free health care at the eve of independence, introduction of the nominal fee in the 1970s and the 1980s full cost recovery, popularly known as the ‘Cash and Carry’ system. Recognizing that direct out-of-pocket payment limited access to health care [17-19], the Government of Ghana declared its intention to abolish the system, and began exploring the feasibility of introducing a national health insurance scheme to be managed at the district level.

The National Health Insurance Act, 2003 (Act 650) established the National Health Insurance Scheme (NHIS) with the aim of increasing access to health care and improving the quality of basic health care services for all citizens, especially the poor and vulnerable. The law establishing the scheme allows for the concurrent operation of District-Wide (Public) Mutual Health Insurance schemes, Private Mutual Health Insurance schemes and Private Commercial Health Insurance schemes [6]. The defined benefit package under the scheme includes inpatient hospital care, outpatient care at primary and secondary levels, and emergency and transfer services. Each client is charged a premium which is renewable annually. Members can have access to services 6 months after registration to curb adverse selection. An exemption policy covers the poor and vulnerable groups; they include the poor, children under the age of 18 years and the elderly above 70 years [6].

### Literature review

Behavioural responses to health utilisation have been studied in various settings across the globe. Whether patients are willing and able to make treatment choices is determined by a range of patient characteristics. Choices may be influenced by social, cultural and religious factors [9,10,13,20]. Andersen–Newman framework is one of the popularly used frameworks for analyzing factors that are linked to patients’ choice of care [15,16]. The model proposes three aspects (predisposing, enabling and needs factors). Predisposing factors include socio-demographic characteristics (age, gender, and education); enabling factors include individual, family and community resources which can include income, costs of care, health insurance, and distance of households from health facilities while need factors refer to condition of an individual’s health such as type and severity of illness [16]. Wealth and income are noted to also affect treatment-seeking behaviour especially for the choice of formal healthcare facilities [9,21-23].

Health insurance is considered an enabling factor as it aims to lower prices at the point of care through risk-sharing. Literature on health insurance and its effect on treatment-seeking behaviour have been vast and varied. The RAND experiment was a randomized trial which focused on the effects of cost-sharing on utilisation and health outcome [23]. The results showed that people delay or forgo healthcare when payments were required at the point of service. In recent times, there have been a number of studies in Asia and Sub Saharan Africa which have looked at the impact of health insurance on treatment choices and health status [24-27]. For instance, Chen et al. [28] found that Taiwan’s NHI greatly increased the utilisation of both outpatient and inpatient services. In a randomised trial using 2,194 households in Ghana, Ansah et al. [29] report an impact on health care-seeking behaviour after removing out-of-pocket payments for health care. Some of these studies show that people tend to move away from informal/self-medication to formal healthcare facilities when they are insured [30]. A study by van den Boom et al. [31] using the 2005/2006 Ghana Living Standards Survey (GLSS) shows that about 80 percent of NHIS members use government or private hospitals compared to 65 percent of the uninsured. Asenso Okyere et al. [18] during the “Cash and Carry” era found that the choice of provider of malaria care was impacted by facility price, travel time, waiting time for treatment and a range of demographic factors (including education, age and sex).

However, there are inefficiencies generated by an increase in demand for care when patients do not face the full price care. Individuals buy health insurance on the basis of several factors. Individuals who are less healthy or suffering from chronic diseases may join the health insurance scheme in order to enjoy its benefits without revealing their true health status. Richer individuals may obtain health insurance for future health benefits [32]. These inherent biases caused by unobservable factors which influence the uptake of health insurance make it difficult to isolate health insurance as the key factor influencing treatment-seeking behaviour [33]. Therefore studies using cross-sectional data can only show association among relationships [34,35].

## Methods

For this paper, a total of 365 adults and children were included in the analyses. Descriptive statistics and multivariate logistic regression analysis were used to describe the characteristics of the sample and to identify factors associated with choice of malaria care. For the bivariate analysis, Pearson’s chi-square test (*X*2) was used to test the association between health insurance ownership and the explanatory variables. For the multivariate analysis, this paper adopts the multinomial logit (MNL) model since the dependent variable is unordered and polychotomous. However, this model requires the ‘Independence of Irrelevant Alternatives’ (IIA) assumption to be satisfied [36]. This property requires that the relative probability of choosing between two alternatives is unaffected by the presence of additional alternatives. To check whether this property holds, a Hausman test procedure was run and the test returned a non significant result (ρ = 0.131), satisfying the assumption [37]. Data analysis was performed using STATA® version 11 and statistical adjustments were made to get robust standard errors since the sampling of respondents in the household involved clustering [38,39].

### Study design

A multi-staged systematic sampling approach to obtain the study population was adopted. Ghana’s 10 administrative regions which are subdivided into 170 districts cut across 3 agro-ecological zones (coastal, forest and savannah). A district was selected in each zone making a total of 3 districts surveyed. A representative household survey was conducted using Enumeration Areas (EAs) based on the 2000 Ghana Population and Housing Census for the selected districts. For each district, 27 EAs, representative of the district were selected. This included both urban and rural communities. Subsequently, 30 households were systematically sampled from the household listing in each EA to obtain the required sample size of 810 in each district; giving a total of 2430 households in all three districts.

The head of household was administered a structured questionnaire. For each household, data was collected on individual and household characteristics (income, education, health insurance status, and treatment-seeking behaviour, illness type, dimensions of quality of care, choice of provider, and reasons for provider choice) as well as community characteristics (whether there was a healthcare facility present in the community). Insured members are described as those who have valid health insurance membership cards in the year of the study. The study took place between January and April, 2011.

### Measures

In the analysis of provider choice for malaria care, the dependant variable is a polychotomous variable reflecting the four healthcare alternatives: i. Informal care; ii. Regional/district hospitals; iii. Public clinic/health centres/CHPS and iv. Private hospitals/clinics. Only individuals reporting illness during the last 4 weeks prior to the study were included and were restricted only to where they first sought care. In total, 1,081 individuals within 358 households reported illness in the last 4 weeks, among 11,089 individuals identified within 2430 households. A total of 1,013 reported seeking care and 68 did not make any attempt to seek care. A total of 365 individuals reported illness type as malaria.

In this paper, informal care includes all individuals who did not seek care from formal health care providers (regional/district hospitals, public clinic/health centres/CHPS and private hospitals/clinics). These informal sources could include seeking care from a drug store (unlicensed chemical shop), or drug peddler without prescription from authorized medical providers or self-prescribed medication based on self-advice.

Among the independent variables include individual characteristics include age, gender, education, health insurance status, nature of illness and travel time to facility (irrespective of mode of transport). The NHIS has an exemption policy in place to ensure that the poor and vulnerable groups in the society have access to healthcare; the exempted groups include the poor, children under the age of 18 years and the elderly (70 years and above). Thus, the three broad age groups (<18 years, 18–69, and 70 years and above) was chosen to reflect this categorization. Household characteristics include a household welfare index as a proxy for household income. Five variables were created with the fifth quintile (highest income group) used as the base group (the omitted variable). The index was constructed using a collection of durable goods owned by the household, materials used in construction of the home, water and sanitation facilities and size of the home [40]. This was calculated using a multivariate technique - Principal Component Analysis (PCA), in which a number of related variables are transformed to a set of uncorrelated variables [41,42]. The resulting asset scores for households were ordered and used to divide households into quintiles, representing their relative wealth with respect to other households in the study. The quintile categories are WQ_1, WQ_2, WQ_3, WQ_4 and WQ_5. A community level characteristic, which is ‘whether there is a health facility in the community’, was also included.

Ethical clearance was sought and granted from the Institutional Review Board (IRB), of the Noguchi Memorial Institute for Medical Research (NMIMR), University of Ghana before the study was done. Study objectives, benefits, risks and the right to refuse participation and confidentiality of responses were explained to participants. Written informed consent was obtained from each participant.

## Results

In total, 365 individuals were available for this sub-analysis in the survey data. Of these individuals, 42 percent were insured and 58 percent uninsured. Table 1 presents the percentage share of individual and household attributes of the insured and non-insured groups. The *P*-values of the differences in the categories is reported in column 4 of Table 1. The uninsured had a higher percentage of individuals with no education (33 percent) compared to 30 percent of the insured. We found that among the insured 64 percent lived in urban areas; among the uninsured 51 percent lived in urban areas. Among the insured, 33 percent were found to be in the highest wealth quintile compared to 16 percent among the uninsured. In the lowest quintile, we find 10 percent of the insured compared to 22 percent of the uninsured. Also, 65 percent of those insured had a health facility in their community compared to 48 percent of the uninsured indicating that proximity to a health facility may influence the demand for insurance.

**Table 1:** **Socio-demographic characteristics of individuals who sought care for malaria by insurance status**

	**Health insurance status**		
	**Insured (%)**	**Uninsured (%)**	P-value*
	**N = 211**	**N = 154**	
**Sex**			
Female	59	53	P = 0.254
Male	41	47	
**Age**			
<18 years	59	75	P = 0.007
18-69 years	37	24	
≥70 years	4	1	
**Education**			
No education	30	33	P = 0.172
Some primary/primary	37	45	
Middle/JSS**	23	17	
Secondary or higher	10	5	
**Residence**			
Urban	64	51	P = 0.015
Rural	36	49	
**Wealth quintiles**			
First (poor)	10	22	P = 0.000
Second	13	27	
Middle	17	19	
Fourth	27	16	
Fifth (non-poor)	33	16	
**Health facility in community**			
Yes	65	48	P = 0.001
No	35	52	

*Chi-square test.

**Juniour Secondary School.

Source: Household data January to April, 2011.

In this section we present a summary of the choice of provider by wealth quintiles, gender, health insurance status, age, settlement type and educational level (Table 2). The *P*-values of the differences in the categories is reported in column 6 of Table 2. In total, 365 of those who reported illness in the last 4 weeks sought some form of care for malaria treatment. Out of those, 58 percent were insured and 42 percent uninsured. Of the total, 71 percent of them sought care from a formal health facility with 62 percent of them insured and 38 percent uninsured. Among the insured, 38 percent consulted at the health centre/clinic, followed by 32 percent who chose regional/district hospital and 14 percent chose private hospital/ clinic whilst 16 percent chose informal care. In the uninsured group, 31 percent consulted at the health centre/clinic, 13 percent regional/district hospital, 8 percent chose private hospital/ clinic whilst 48 percent chose informal care. These results were significant at 1 percent level, showing clearly that a larger share of the uninsured individuals chose informal care than the insured when seeking treatment for malaria.

**Table 2:** **Choice of care among individuals seeking care by insurance status, demographic and socio-economic characteristics and type of illness**

**Variable**	**Regional/District hospital (%)**	**Private hospital/clinic (%)**	**Public health centre/clinic (%)**	**Informal care (%)**	P-value*
	**N = 85 (24)**	**N = 41 (12)**	**N = 124 (35)**	**N = 102(29)**	
**Insurance status**					
Insured	32	14	38	16	P = 0.000
Uninsured	13	8	31	48	
**Wealth quintiles**					
First (poor)	16	14	39	31	P = 0.141
Second	13	11	37	39	
Third	27	10	37	26	
Fourth	21	13	34	33	
Fifth (non poor)	36	12	32	20	
**Sex**					
Male	26	10	33	31	P = 0.667
Female	23	13	37	27	
**Age**					
<18	22	10	37	31	P = 0.314
18-69	31	14	32	23	
70+	24	12	35	29	
**Education**					
No education	20	16	29	35	P = 0.018
Some primary	29	8	39	24	
Middle/JSS**	35	10	40	15	
Secondary & above	14	0	38	48	
**Residence**					
Urban	30	11	34	25	P = 0.026
Rural	16	12	38	34	

*Chi-square test.

**Juniour Secondary School.

Source: Household data January to April, 2011.

For choice of health provider by education level, the results show that 35 percent of those with no education and 48 percent of those with secondary education and above chose informal care. This is at 5 percent significant level (Table 2). However those who had secondary level or above education were 7 percent of the total sample which explains the large proportion of those seeking care from informal care in this category. Also, a higher proportion of urban dwellers sought care from regional/district hospitals compared to rural (30 versus 16 percent).

Individuals were asked to give one main reason influencing their choice of a healthcare provider. Results indicate that of the total sample, proximity to the health facility was the most frequent reason for choice of provider. For those who chose informal care 68 percent gave proximity as the main reason with about 65 percent of those who chose health centres/clinics also citing proximity was the main reason of choice. By health insurance status, 62 percent of the uninsured were influenced by proximity to source of care compared to 46 percent of the insured. These differences are statistically significant at 1% level. This shows the how proximity to health facilities influences the choice of provider.

This section presents the results from the MNL estimation in Table 3. A description of the variables and their summary statistics is shown in Table A in the Appendix. We present the relative risk ratios (RRR) for each type of health facility chosen. The RRR is interpreted as the relative probability of choosing alternative health provider to informal care (the comparison group for the MNL estimation) for individuals with a particular characteristic, compared to the comparison group. For each of the independent variables, the comparison groups have been indicated in Table A in the Appendix.

**Table 3:** **Relative risk ratios estimation showing the probability of choice of healthcare in last 4 weeks, with wealth quintiles**

**Variable**	**Regional/District hospital**	**Private hospital/clinic**	**Public health centre/clinic**
**Health insurance**	5.76*** [2.54]	7.17*** [4.15]	4.85*** [1.89]
**WQ_1**	0.25 [0.22]	0.45 [0.47]	1.07 [0.73]
**WQ_2**	0.21** [0.15]	0.54 [0.47]	0.95 [0.56]
**WQ_3**	0.74 [0.16]	0.44 [0.38]	1.15 [0.67]
**WQ_4**	0.28** [0.14]	0.39 [0.26]	0.43 [0.24]
**Primary educ**	2.49* [1.23]	0.98 [0.58]	2.30** [1.02]
**JSS educ**	2.33 [1.54]	0.77 [0.58]	3.94** [2.21]
**Secondary educ**	0.30 [0.24]	4.63*** [3.08]	0.99 [0.64]
**Male**	1.12 [0.44]	0.78 [0.39]	0.89 [0.32]
**HFAC in community**	1.71 [0.85]	1.09 [0.61]	1.64 [0.69]
**<18 years**	0.48 [0.26]	0.36* [0.22]	1.12 [0.57]
**>70 years**	0.17* [0.17]	0.71 [0.68]	0.46 [0.39]
**Time to facility**	1.05** [0.03]	1.05** [0.03]	1.04* [0.01]

Robust standard errors are in parentheses.

*Significant at 10%.

**Significant at 5%.

***Significant at 1%.

Source: Household data January to April, 2011.

The findings show that health insurance is a significant determinant of choice of care. For instance those insured are 6 times more likely to choose regional/district hospitals: 5 times more likely to choose health centres/clinics and 7 times more likely to choose private hospitals/clinics over informal care when compared to those uninsured and this is significant at 1 percent (Table 3). Using the wealth quintiles, significance is only seen with the choice of regional/district hospitals. The findings show that compared to the fifth quintile (non poor), those in the second and fourth quintiles are less likely to choose regional/district hospitals (5 percent significant level). There is no significant difference between quintiles in the other choice options. This implies that the wealthier you are the more able you are to seek care from expensive medical care.

Age, gender, and having a health facility in community are not significant determinants of choice. The education variable is not significant with the choice of care from the regional/district hospitals but highly significant in the choice of private hospitals/clinics and health centres/clinics. For instance, individuals with secondary education or more are 4 times more likely to choose private hospitals/clinics over informal care, when compared to those with no education. This is significant at 1 percent and similar results are for the choice of public health centres/clinics at 5 percent significance level. The results may imply that higher education may enable individuals to access information about the health risks of using informal care for malaria treatment than those with no education.

Travel time to facility irrespective of mode of transportation is a significant determinant of choice of care (5 percent significance level) for choices of regional/district hospitals and private hospitals/clinics and 10 percent significant level for health centres/clinics relative to informal care. For the exposure status to be related to the outcome, the relative risk must differ from 1. However, the relative risk is 1.05 implies only a small difference in the way a unit increment in travel time (measured in minutes) affects the two groups.

## Discussion

This paper seeks to investigate factors that affect the care-seeking behaviour of individuals when ill with malaria in Ghana under the National Health Insurance Scheme (NHIS). One of the main objectives of the NHIS is to ensure access to health care. Our findings suggest that health insurance status and travel time to facility are the two main determinants of care-seeking behaviour. For the predisposed factors, age and gender are not significant determinants but educational status is a significant determinant for choice of private hospitals/clinic and health centres. A plausible reason could be that the more educated individuals were more likely to seek care from these facilities than those with no formal education.

The study shows that insurance status influences the choice of healthcare sought by individuals with malaria. However, what is also worth noting is the fact that coverage of the scheme remains below 40 percent of the Ghanaian population. This means that a large number of uninsured therefore have limited access to care in formal healthcare facilities. Resorting to informal sources of care for the treatment of malaria predisposes patients to poor quality of malaria diagnosis and treatment. In Ghana, some community members self-medicate as a result of the high cost of care [18].

Household wealth status was an important determinant of care for only regional/district hospitals. The hierarchical nature of the health system in Ghana would suggest that regional/district hospitals are quite a distance for a number of communities especially in the rural areas. Therefore, there is an additional cost incurred through transportation costs as well as opportunity cost if care is sought from such facilities. Proximity to providers was also a key reason reported by care seekers and not surprisingly the results show that a higher proportion of the uninsured cite this reason compared to those insured. This is consistent with other studies which demonstrate preferences for health care providers in the same locality or in locations easier and affordable to reach [43]. This is also further explained by the fact that health insurance status was lower among the poorer quintiles. This is consistent with previous studies in Ghana [44-47].

We also find that public health centres were the choice of care by both the insured and uninsured (38 percent versus 31 percent). Again, the hierarchical organisation of the health sector, suggests that public health centres would be the first point of care since they are geographically closer to most households. Here again we may consider the issue of proximity to care or the possible explanation that treatment costs at public health centres are affordable even without insurance.

For instance, van Doorslaer et al. [48] note that out-of-pocket expenditures on healthcare is a considerable share of household income. Since this study did not expand to include the cost of care, the effect of this indicator is unknown and remains a limitation in this study. Also, the cross-sectional nature of the study precludes any claim of causal effect of health insurance on care-seeking behaviour for malaria treatment in Ghana.

## Conclusion

Household surveys such as this allows the analysis of the factors that affect the different health care provider choices. Understanding these in relation to malaria treatment and in the larger sphere of the health care delivery system is of considerable policy significance. We analyze the care-seeking behaviour of individuals reporting malaria in the framework of the NHIS in Ghana. The results demonstrate that health insurance is a significant determinant of choice of provider. The uninsured compared to the insured are more likely to choose informal health facilities than seek care from formal healthcare providers.

There have been issues raised about the equitable nature of Ghana’s health insurance scheme. The positive effect of the NHIS scheme in making malaria care more affordable seems to be the reserve of individuals with higher wealth status. Notably, some vulnerable groups especially the poor are unable to benefit from the exemption scheme package intended for them. Exposing the vulnerable and other uninsured groups to the past inadequacies of the ‘Cash and Carry’ system does not augur well for the health system. Health policies that seek to promote equity through better access to health facilities, as well as government policies that ensure access to good roads especially in rural areas should be encouraged.
